# 2,6-Diethyl­anilinium perchlorate

**DOI:** 10.1107/S1600536810004654

**Published:** 2010-02-13

**Authors:** Wajda Smirani Sta, Mohamed Rzaigui, Salem S. Al-Deyab

**Affiliations:** a Laboratoire de Chimie des Matériaux, Faculté des Sciences de Bizerte, 7021 Zarzouna, Bizerte, Tunisia; b Petrochemical Research Chair, College of Science, King Saud University, Riyadh, Saudi Arabia

## Abstract

The asymmetric unit of the title mol­ecular salt, C_10_H_16_N^+^·ClO_4_^−^, contains two cations and two anions. The atoms of one of the ethyl side chains of one of the cations are disordered over two sets of sites in a 0.531 (13):0.469 (13) ratio. In the crystal, the components are linked by N—H⋯O and bifurcated N—H⋯(O,O) hydrogen bonds and weaker C—H⋯O inter­actions, such that the organic cations alternate with the perchlorate anions, forming ribbons in the *a*-axis direction.

## Related literature

For background to the physical properties and potential applications of mol­ecular salts, see: Czarnecki *et al.* (1994 ▶); Mylrajan & Srinivasan (1991 ▶); Toumi Akriche *et al.* (2010 ▶); Xiao *et al.* (2005 ▶). For the graph-set notation of hydrogen-bond networks, see: Bernstein *et al.* (1995 ▶). 
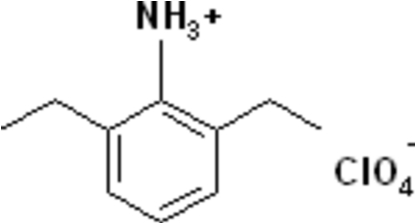


## Experimental

### 

#### Crystal data

C_10_H_16_N^+^·ClO_4_^−^
*M* *_r_* = 249.69
Monoclinic, 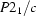

*a* = 15.105 (3) Å
*b* = 21.192 (5) Å
*c* = 7.718 (6) Å
β = 98.10 (3)°
*V* = 2446 (2) Å^3^
*Z* = 8
Ag *K*α radiation
λ = 0.56085 Å
μ = 0.17 mm^−1^
*T* = 293 K
0.50 × 0.40 × 0.20 mm

#### Data collection

Enraf–Nonius TurboCAD-4 diffractometer
15750 measured reflections
11941 independent reflections
2954 reflections with *I* > 2σ(*I*)
*R*_int_ = 0.052
2 standard reflections every 120 min  intensity decay: 5%

#### Refinement

*R*[*F* ^2^ > 2σ(*F* ^2^)] = 0.089
*wR*(*F* ^2^) = 0.278
*S* = 0.91
11941 reflections
304 parameters
H-atom parameters not refined
Δρ_max_ = 0.36 e Å^−3^
Δρ_min_ = −0.36 e Å^−3^

### 

Data collection: *CAD-4 EXPRESS* (Enraf–Nonius, 1994 ▶); cell refinement: *CAD-4 EXPRESS*; data reduction: *XCAD4* (Harms & Wocadlo, 1995 ▶); program(s) used to solve structure: *SHELXS97* (Sheldrick, 2008 ▶); program(s) used to refine structure: *SHELXL97* (Sheldrick, 2008 ▶); molecular graphics: *ORTEP-3* (Farrugia, 1997 ▶); software used to prepare material for publication: *WinGX* (Farrugia, 1999 ▶).

## Supplementary Material

Crystal structure: contains datablocks I, New_Global_Publ_Block. DOI: 10.1107/S1600536810004654/hb5332sup1.cif

Structure factors: contains datablocks I. DOI: 10.1107/S1600536810004654/hb5332Isup2.hkl

Additional supplementary materials:  crystallographic information; 3D view; checkCIF report

## Figures and Tables

**Table 1:** Hydrogen-bond geometry (Å, °)

*D*—H⋯*A*	*D*—H	H⋯*A*	*D*⋯*A*	*D*—H⋯*A*
N1—H1*A*⋯O6	0.89	2.41	3.029 (4)	127
N1—H1*B*⋯O4	0.89	2.27	3.043 (5)	146
N1—H1*B*⋯O2^i^	0.89	2.48	2.889 (4)	109
N1—H1*C*⋯O3^ii^	0.89	2.10	2.935 (4)	157
N1—H1*C*⋯O2^i^	0.89	2.58	2.889 (4)	101
N2—H2*A*⋯O1	0.89	2.17	2.971 (4)	149
N2—H2*B*⋯O5	0.89	2.39	2.875 (4)	114
N2—H2*B*⋯O7^iii^	0.89	2.24	2.991 (4)	141
N2—H2*C*⋯O7^iv^	0.89	2.03	2.805 (3)	144
C3—H3⋯O3^v^	0.93	2.60	3.321 (5)	134
C13—H13⋯O8^vi^	0.93	2.49	3.418 (6)	172

Symmetry codes: (i) 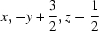; (ii) 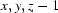; (iii) 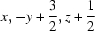; (iv) 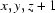; (v) 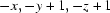; (vi) 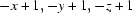.

**Fig. 1.**
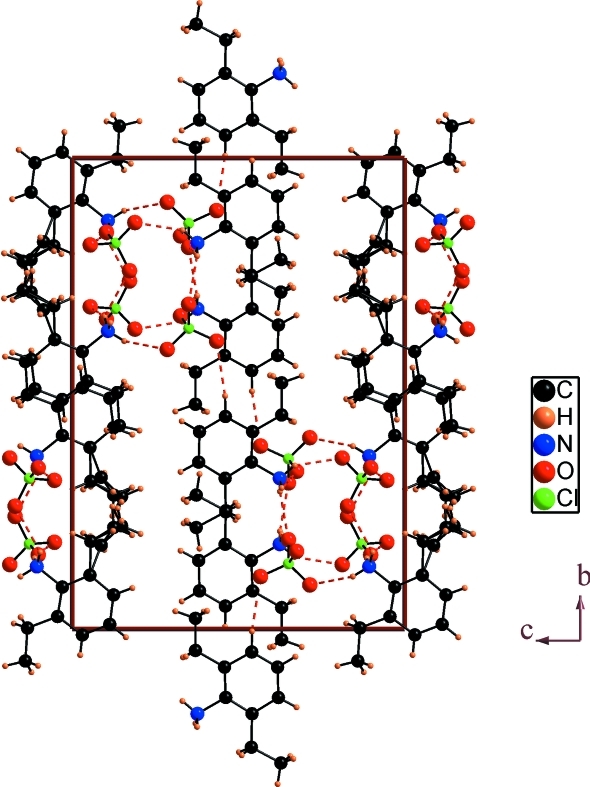
A view of the packing of (I) along the a axis.

**Fig. 2.**
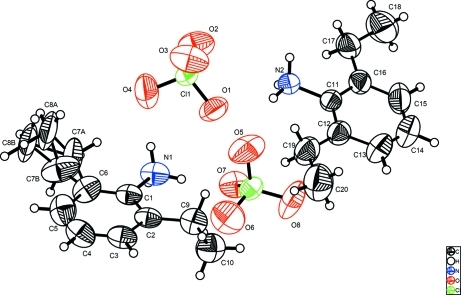
The molecular structure of (I) with displacement ellipsoids for non-H atoms drawn at the 30% probability level.

## supplementary crystallographic information

### Comment

A special attention is focused on the synthesis of hybrid class of inorganic-organic materials because of their interesting architectures and wide variety of physical properties. Organic substructure of these compounds is usually responsible for their molecular hyperpolarizability, electric permittivity and spontaneous polarization. The inorganic part determines thermal and mechanical stability of the crystals. The combination of these various features attributed to both organic and inorganic substructures may lead to interesting materials (Xiao *et al.*, 2005). In particular, the association of the perchlorate anions to organic molecules could lead to materials having phase transitions, non-symmetric structures, characteristic H-bonds (Czarnecki *et al.*, 1994; Mylrajan & Srinivasan, 1991). Among the crystals comprising perchlorate anions, the most interesting are non-centrosymmetric, due to non linear optical(ONL) properties. A pronounced second harmonic generation was found in L-leucinium perchlorate (SHG efficiency d eff= 0.44 x d eff KDP). In this paper, we report single-crystal X-ray study of 2,6-diethylanilinium perchlorate (I). Crystal structure of this latter is depicted in the figure 1. The asymmetric unit, built of two 2,6-diethylanilinium cations and two perchlorate anions, has the geometrical configuration shown in the figure 2. These four components establish between them H-bonds to form a tetra-membered ring. This ring form two slightly corrugated ribbons parallel to the a direction at y = 1/4 and 3/4. Each ribbon is built of an alternance of both inorganic and organic entities.

The first Cl(1)O_4_^-^ is surrounded by three N(1)H_3_^+^cations, to build a ribbon extended in the a direction, and generating R_2_^4^(8) graph-set motifs.Whereas the second Cl(2)O_4_^-^, surrounded by two N(2)H_3_^+^cations, leads to R_2_^4^(10) graph-set motifs which form another ribbon extended in the same direction. Both parallel ribbons are attached together by N—H···O hydrogen bonds. The organic molecules are anchored on these ribbons so that to leave spacious channels (13.9 Å x 3.3 Å) parallel to the a axis. The Cl—O distances indicate rather slight distortion of the two perchlorate anions from the tetrahedral symmetry. The shortest Cl—O bond equals to 1.384 (3) Å, whereas the longest one to 1.444 (3) Å, the angles vary from 105.6 (2)° to 111.2 (2)° that are standard values for perchlorate ions (Toumi Akriche, S. *et al.* 2010). In this organisation, the components display different interactions (electrostatic, H-bonds, Van derWalls) to keep the three-dimensional network stability.

### Experimental

An ethalonic 2,6-diethylaniline solution (5 mmol, in 5 ml) was added to an aqueous perchloric acid solution (0.5 M, 10 ml) at room temperature (293 K). Slow evaporation of the obtained mixture led to the formation of small crystals of the title compound.  These were recrystallised from a mixture of water / ethanol (80% / 20%) to yield colourless blocks of (I).

### Refinement

All H atoms were positioned geometrically (C—H = 0.93–0.97Å, N—H = 0.89Å) and refined as riding with *U*_iso_(H) = 1.2U_eq_(carrier) or 1.5U_eq_(methyl C).

### Crystal data

**Table:**

C_10_H_16_N^+^·ClO_4_^−^	*F*(000) = 1056
*M**_r_* = 249.69	*D*_x_ = 1.356 Mg m^−^^3^
Monoclinic, *P*2_1_/*c*	Ag *K*α radiation, λ = 0.56085 Å
Hall symbol: -P 2ybc	Cell parameters from 25 reflections
*a* = 15.105 (3) Å	θ = 9–11°
*b* = 21.192 (5) Å	µ = 0.17 mm^−^^1^
*c* = 7.718 (6) Å	*T* = 293 K
β = 98.10 (3)°	Block, colourless
*V* = 2446 (2) Å^3^	0.50 × 0.40 × 0.20 mm
*Z* = 8	

### Data collection

**Table:**

Enraf–Nonius TurboCAD-4 diffractometer	*R*_int_ = 0.052
Radiation source: fine-focus sealed tube	θ_max_ = 28.0°, θ_min_ = 2.2°
graphite	*h* = −25→5
non–profiled ω scans	*k* = −35→0
15750 measured reflections	*l* = −12→12
11941 independent reflections	2 standard reflections every 120 min
2954 reflections with *I* > 2σ(*I*)	intensity decay: 5%

### Refinement

**Table:**

Refinement on *F*^2^	Primary atom site location: structure-invariant direct methods
Least-squares matrix: full	Secondary atom site location: difference Fourier map
*R*[*F*^2^ > 2σ(*F*^2^)] = 0.089	Hydrogen site location: inferred from neighbouring sites
*wR*(*F*^2^) = 0.278	H-atom parameters not refined
*S* = 0.91	*w* = 1/[σ^2^(*F*_o_^2^) + (0.1095*P*)^2^ + ] where *P* = (*F*_o_^2^ + 2*F*_c_^2^)/3
11941 reflections	(Δ/σ)_max_ < 0.001
304 parameters	Δρ_max_ = 0.36 e Å^−^^3^
0 restraints	Δρ_min_ = −0.36 e Å^−^^3^

### Special details

**Table:**

Geometry. All esds (except the esd in the dihedral angle between two l.s. planes) are estimated using the full covariance matrix. The cell esds are taken into account individually in the estimation of esds in distances, angles and torsion angles; correlations between esds in cell parameters are only used when they are defined by crystal symmetry. An approximate (isotropic) treatment of cell esds is used for estimating esds involving l.s. planes.
Refinement. Refinement of *F*^2^ against ALL reflections. The weighted *R*-factor wR and goodness of fit *S* are based on *F*^2^, conventional *R*-factors *R* are based on *F*, with *F* set to zero for negative *F*^2^. The threshold expression of *F*^2^ > σ(*F*^2^) is used only for calculating *R*-factors(gt) etc. and is not relevant to the choice of reflections for refinement. *R*-factors based on *F*^2^ are statistically about twice as large as those based on *F*, and *R*- factors based on ALL data will be even larger.

### Fractional atomic coordinates and isotropic or equivalent isotropic displacement parameters (Å^2^)

**Table:**

	*x*	*y*	*z*	*U*_iso_*/*U*_eq_	Occ. (<1)
C7A	−0.0545 (10)	0.6898 (8)	0.139 (3)	0.094 (7)	0.469 (13)
H7A1	0.0024	0.7060	0.1960	0.112*	0.469 (13)
H7A2	−0.0600	0.7012	0.0165	0.112*	0.469 (13)
C7B	−0.0813 (12)	0.6774 (8)	0.089 (2)	0.106 (6)	0.531 (13)
H7B1	−0.0280	0.7030	0.1180	0.127*	0.531 (13)
H7B2	−0.0885	0.6726	−0.0373	0.127*	0.531 (13)
C8A	−0.1146 (10)	0.7157 (9)	0.204 (3)	0.126 (4)	0.469 (13)
H8A1	−0.1124	0.7604	0.1839	0.188*	0.469 (13)
H8A2	−0.1072	0.7076	0.3273	0.188*	0.469 (13)
H8A3	−0.1713	0.6995	0.1504	0.188*	0.469 (13)
C8B	−0.1498 (9)	0.7177 (8)	0.116 (3)	0.126 (4)	0.531 (13)
H8B1	−0.1429	0.7570	0.0571	0.188*	0.531 (13)
H8B2	−0.1481	0.7251	0.2387	0.188*	0.531 (13)
H8B3	−0.2061	0.6990	0.0695	0.188*	0.531 (13)
N2	0.37636 (18)	0.68014 (13)	0.7366 (3)	0.0541 (7)	
H2A	0.3312	0.6531	0.7121	0.081*	
H2B	0.3754	0.7078	0.6497	0.081*	
H2C	0.3709	0.7005	0.8355	0.081*	
O3	0.1026 (2)	0.65755 (15)	0.8694 (4)	0.1012 (11)	
O1	0.18825 (19)	0.63681 (13)	0.6498 (4)	0.0866 (9)	
O7	0.3499 (2)	0.68975 (12)	0.0880 (3)	0.0791 (8)	
N1	0.1070 (2)	0.63278 (14)	0.2448 (4)	0.0658 (8)	
H1A	0.1561	0.6116	0.2870	0.099*	
H1B	0.0970	0.6629	0.3201	0.099*	
H1C	0.1141	0.6502	0.1428	0.099*	
O2	0.1721 (2)	0.73912 (12)	0.7426 (5)	0.1005 (11)	
O5	0.3253 (3)	0.66497 (17)	0.3658 (4)	0.1196 (14)	
O8	0.4341 (2)	0.61129 (16)	0.2440 (6)	0.1222 (14)	
O6	0.2856 (2)	0.59355 (16)	0.1434 (5)	0.1159 (12)	
O4	0.0539 (2)	0.6843 (2)	0.5835 (5)	0.1218 (13)	
Cl1	0.12968 (6)	0.68032 (4)	0.71443 (11)	0.0502 (2)	
Cl2	0.35009 (6)	0.63848 (4)	0.21107 (12)	0.0580 (3)	
C1	0.0299 (2)	0.58913 (16)	0.2194 (4)	0.0544 (9)	
C3	−0.0302 (3)	0.48757 (18)	0.2325 (5)	0.0674 (11)	
H3	−0.0236	0.4448	0.2587	0.081*	
C2	0.0446 (3)	0.52653 (16)	0.2615 (4)	0.0562 (9)	
C4	−0.1126 (3)	0.5099 (2)	0.1672 (5)	0.0786 (12)	
H4	−0.1612	0.4825	0.1497	0.094*	
C6	−0.0529 (3)	0.6145 (2)	0.1543 (6)	0.0713 (11)	
C9	0.1350 (3)	0.50228 (19)	0.3346 (5)	0.0723 (11)	
H9A	0.1554	0.5253	0.4414	0.087*	
H9B	0.1758	0.5118	0.2518	0.087*	
C10	0.1413 (3)	0.43215 (19)	0.3754 (6)	0.0896 (14)	
H10A	0.2019	0.4216	0.4210	0.134*	
H10B	0.1231	0.4085	0.2703	0.134*	
H10C	0.1030	0.4221	0.4606	0.134*	
C5	−0.1239 (3)	0.5723 (2)	0.1274 (6)	0.0850 (13)	
H5	−0.1804	0.5869	0.0813	0.102*	
C11	0.4615 (2)	0.64544 (17)	0.7574 (4)	0.0548 (9)	
C16	0.5406 (3)	0.6799 (2)	0.7952 (5)	0.0634 (10)	
C12	0.4577 (3)	0.58089 (18)	0.7432 (5)	0.0647 (10)	
C20	0.3702 (4)	0.4774 (3)	0.7116 (8)	0.125 (2)	
H20A	0.3098	0.4624	0.6854	0.187*	
H20B	0.3956	0.4630	0.8258	0.187*	
H20C	0.4049	0.4616	0.6260	0.187*	
C13	0.5396 (3)	0.5487 (2)	0.7667 (6)	0.0885 (14)	
H13	0.5405	0.5049	0.7598	0.106*	
C19	0.3704 (3)	0.5457 (2)	0.7082 (7)	0.0876 (14)	
H19A	0.3333	0.5600	0.7930	0.105*	
H19B	0.3411	0.5587	0.5938	0.105*	
C17	0.5385 (3)	0.7511 (2)	0.8118 (6)	0.0765 (11)	
H17A	0.5014	0.7616	0.9005	0.092*	
H17B	0.5093	0.7682	0.7017	0.092*	
C15	0.6199 (3)	0.6456 (3)	0.8166 (6)	0.0883 (14)	
H15	0.6745	0.6663	0.8425	0.106*	
C14	0.6174 (3)	0.5811 (3)	0.7994 (7)	0.0945 (15)	
H14	0.6710	0.5589	0.8106	0.113*	
C18	0.6274 (4)	0.7845 (3)	0.8577 (8)	0.120 (2)	
H18A	0.6176	0.8292	0.8628	0.180*	
H18B	0.6649	0.7755	0.7700	0.180*	
H18C	0.6562	0.7699	0.9694	0.180*	

### Atomic displacement parameters (Å^2^)

**Table:**

	*U*^11^	*U*^22^	*U*^33^	*U*^12^	*U*^13^	*U*^23^
C7A	0.044 (6)	0.062 (9)	0.168 (16)	0.000 (6)	−0.007 (7)	0.039 (9)
C7B	0.119 (14)	0.088 (8)	0.130 (11)	−0.023 (9)	0.083 (11)	−0.023 (8)
C8A	0.050 (8)	0.135 (6)	0.187 (16)	0.045 (7)	−0.002 (6)	0.010 (9)
C8B	0.050 (8)	0.135 (6)	0.187 (16)	0.045 (7)	−0.002 (6)	0.010 (9)
N2	0.0530 (17)	0.0584 (16)	0.0511 (16)	0.0024 (14)	0.0081 (14)	−0.0052 (13)
O3	0.146 (3)	0.103 (2)	0.0636 (17)	−0.043 (2)	0.0447 (19)	−0.0017 (16)
O1	0.0757 (19)	0.0764 (18)	0.114 (2)	0.0053 (15)	0.0331 (18)	−0.0259 (16)
O7	0.107 (2)	0.0746 (17)	0.0590 (15)	−0.0023 (16)	0.0237 (16)	0.0112 (13)
N1	0.077 (2)	0.0641 (19)	0.0620 (18)	−0.0194 (16)	0.0293 (16)	−0.0043 (14)
O2	0.128 (3)	0.0523 (16)	0.129 (3)	−0.0247 (17)	0.044 (2)	−0.0107 (16)
O5	0.181 (4)	0.136 (3)	0.0490 (16)	0.062 (3)	0.041 (2)	0.0098 (17)
O8	0.073 (2)	0.082 (2)	0.216 (4)	0.0294 (18)	0.034 (2)	0.020 (2)
O6	0.108 (3)	0.091 (2)	0.149 (3)	−0.036 (2)	0.022 (2)	−0.002 (2)
O4	0.083 (2)	0.165 (3)	0.107 (3)	0.030 (2)	−0.020 (2)	−0.002 (2)
Cl1	0.0526 (5)	0.0464 (4)	0.0535 (5)	−0.0003 (4)	0.0142 (4)	−0.0012 (4)
Cl2	0.0594 (6)	0.0571 (5)	0.0608 (5)	0.0063 (5)	0.0198 (4)	0.0041 (4)
C1	0.062 (2)	0.057 (2)	0.0494 (18)	−0.0151 (18)	0.0236 (17)	−0.0073 (15)
C3	0.086 (3)	0.056 (2)	0.064 (2)	−0.022 (2)	0.021 (2)	−0.0045 (17)
C2	0.072 (3)	0.055 (2)	0.0449 (18)	−0.0150 (18)	0.0206 (18)	−0.0058 (15)
C4	0.076 (3)	0.091 (3)	0.071 (3)	−0.033 (3)	0.017 (2)	0.001 (2)
C6	0.076 (3)	0.068 (3)	0.078 (3)	−0.004 (2)	0.036 (2)	0.009 (2)
C9	0.076 (3)	0.072 (3)	0.071 (3)	−0.010 (2)	0.017 (2)	0.004 (2)
C10	0.104 (4)	0.075 (3)	0.089 (3)	0.004 (3)	0.012 (3)	0.012 (2)
C5	0.065 (3)	0.111 (4)	0.082 (3)	−0.006 (3)	0.020 (2)	0.013 (3)
C11	0.060 (2)	0.067 (2)	0.0380 (17)	0.0130 (19)	0.0068 (16)	−0.0003 (15)
C16	0.058 (2)	0.079 (3)	0.053 (2)	0.007 (2)	0.0057 (18)	−0.0018 (18)
C12	0.068 (3)	0.065 (2)	0.062 (2)	0.009 (2)	0.009 (2)	0.0027 (18)
C20	0.101 (4)	0.105 (4)	0.163 (6)	0.001 (3)	−0.001 (4)	0.017 (4)
C13	0.082 (3)	0.075 (3)	0.110 (4)	0.025 (3)	0.020 (3)	0.007 (3)
C19	0.077 (3)	0.063 (3)	0.121 (4)	0.010 (2)	0.006 (3)	−0.002 (2)
C17	0.060 (3)	0.090 (3)	0.078 (3)	−0.007 (2)	0.007 (2)	−0.013 (2)
C15	0.057 (3)	0.114 (4)	0.091 (3)	0.009 (3)	−0.001 (2)	0.004 (3)
C14	0.066 (3)	0.100 (4)	0.119 (4)	0.024 (3)	0.017 (3)	0.009 (3)
C18	0.095 (4)	0.118 (4)	0.145 (5)	−0.031 (3)	0.010 (4)	−0.024 (4)

### Geometric parameters (Å, °)

**Table:**

C7A—C8A	1.22 (3)	C2—C9	1.493 (5)
C7A—C6	1.599 (17)	C4—C5	1.363 (6)
C7A—H7A1	0.9700	C4—H4	0.9300
C7A—H7A2	0.9700	C6—C5	1.390 (6)
C7B—C8B	1.38 (2)	C9—C10	1.520 (5)
C7B—C6	1.468 (19)	C9—H9A	0.9700
C7B—H7B1	0.9700	C9—H9B	0.9700
C7B—H7B2	0.9700	C10—H10A	0.9600
C8A—H8A1	0.9600	C10—H10B	0.9600
C8A—H8A2	0.9600	C10—H10C	0.9600
C8A—H8A3	0.9600	C5—H5	0.9300
C8B—H8B1	0.9600	C11—C12	1.373 (5)
C8B—H8B2	0.9600	C11—C16	1.395 (5)
C8B—H8B3	0.9600	C16—C15	1.392 (6)
N2—C11	1.471 (4)	C16—C17	1.515 (6)
N2—H2A	0.8900	C12—C13	1.402 (5)
N2—H2B	0.8900	C12—C19	1.506 (6)
N2—H2C	0.8900	C20—C19	1.446 (6)
O3—Cl1	1.404 (3)	C20—H20A	0.9600
O1—Cl1	1.416 (3)	C20—H20B	0.9600
O7—Cl2	1.443 (3)	C20—H20C	0.9600
N1—C1	1.478 (4)	C13—C14	1.354 (6)
N1—H1A	0.8900	C13—H13	0.9300
N1—H1B	0.8900	C19—H19A	0.9700
N1—H1C	0.8900	C19—H19B	0.9700
O2—Cl1	1.404 (3)	C17—C18	1.515 (6)
O5—Cl2	1.417 (3)	C17—H17A	0.9700
O8—Cl2	1.385 (3)	C17—H17B	0.9700
O6—Cl2	1.409 (3)	C15—C14	1.374 (6)
O4—Cl1	1.418 (3)	C15—H15	0.9300
C1—C2	1.376 (5)	C14—H14	0.9300
C1—C6	1.389 (5)	C18—H18A	0.9600
C3—C4	1.360 (6)	C18—H18B	0.9600
C3—C2	1.391 (5)	C18—H18C	0.9600
C3—H3	0.9300		
			
C8A—C7A—C6	115.0 (16)	C5—C6—C7B	110.6 (8)
C8A—C7A—H7A1	108.5	C1—C6—C7A	114.6 (6)
C6—C7A—H7A1	108.5	C5—C6—C7A	128.9 (7)
C8A—C7A—H7A2	108.5	C7B—C6—C7A	21.4 (10)
C6—C7A—H7A2	108.5	C2—C9—C10	116.3 (4)
H7A1—C7A—H7A2	107.5	C2—C9—H9A	108.2
C8B—C7B—C6	134.4 (14)	C10—C9—H9A	108.2
C8B—C7B—H7B1	103.6	C2—C9—H9B	108.2
C6—C7B—H7B1	103.6	C10—C9—H9B	108.2
C8B—C7B—H7B2	103.6	H9A—C9—H9B	107.4
C6—C7B—H7B2	103.6	C9—C10—H10A	109.5
H7B1—C7B—H7B2	105.3	C9—C10—H10B	109.5
C7A—C8A—H8A1	109.5	H10A—C10—H10B	109.5
C7A—C8A—H8A2	109.5	C9—C10—H10C	109.5
H8A1—C8A—H8A2	109.5	H10A—C10—H10C	109.5
C7A—C8A—H8A3	109.5	H10B—C10—H10C	109.5
H8A1—C8A—H8A3	109.5	C4—C5—C6	121.4 (5)
H8A2—C8A—H8A3	109.5	C4—C5—H5	119.3
C7B—C8B—H8B1	109.5	C6—C5—H5	119.3
C7B—C8B—H8B2	109.5	C12—C11—C16	124.3 (4)
H8B1—C8B—H8B2	109.5	C12—C11—N2	117.6 (3)
C7B—C8B—H8B3	109.5	C16—C11—N2	118.1 (3)
H8B1—C8B—H8B3	109.5	C15—C16—C11	116.7 (4)
H8B2—C8B—H8B3	109.5	C15—C16—C17	122.5 (4)
C11—N2—H2A	109.5	C11—C16—C17	120.8 (3)
C11—N2—H2B	109.5	C11—C12—C13	116.7 (4)
H2A—N2—H2B	109.5	C11—C12—C19	122.3 (4)
C11—N2—H2C	109.5	C13—C12—C19	121.0 (4)
H2A—N2—H2C	109.5	C19—C20—H20A	109.5
H2B—N2—H2C	109.5	C19—C20—H20B	109.5
C1—N1—H1A	109.5	H20A—C20—H20B	109.5
C1—N1—H1B	109.5	C19—C20—H20C	109.5
H1A—N1—H1B	109.5	H20A—C20—H20C	109.5
C1—N1—H1C	109.5	H20B—C20—H20C	109.5
H1A—N1—H1C	109.5	C14—C13—C12	120.3 (4)
H1B—N1—H1C	109.5	C14—C13—H13	119.9
O3—Cl1—O2	110.8 (2)	C12—C13—H13	119.9
O3—Cl1—O1	110.4 (2)	C20—C19—C12	119.8 (4)
O2—Cl1—O1	109.79 (19)	C20—C19—H19A	107.4
O3—Cl1—O4	108.9 (2)	C12—C19—H19A	107.4
O2—Cl1—O4	111.2 (2)	C20—C19—H19B	107.4
O1—Cl1—O4	105.6 (2)	C12—C19—H19B	107.4
O8—Cl2—O6	110.8 (2)	H19A—C19—H19B	106.9
O8—Cl2—O5	110.8 (3)	C16—C17—C18	117.2 (4)
O6—Cl2—O5	109.2 (2)	C16—C17—H17A	108.0
O8—Cl2—O7	110.6 (2)	C18—C17—H17A	108.0
O6—Cl2—O7	109.1 (2)	C16—C17—H17B	108.0
O5—Cl2—O7	106.27 (19)	C18—C17—H17B	108.0
C2—C1—C6	124.4 (3)	H17A—C17—H17B	107.2
C2—C1—N1	118.4 (3)	C14—C15—C16	119.7 (5)
C6—C1—N1	117.3 (3)	C14—C15—H15	120.1
C4—C3—C2	122.2 (4)	C16—C15—H15	120.1
C4—C3—H3	118.9	C13—C14—C15	122.3 (5)
C2—C3—H3	118.9	C13—C14—H14	118.8
C1—C2—C3	115.8 (4)	C15—C14—H14	118.8
C1—C2—C9	121.9 (3)	C17—C18—H18A	109.5
C3—C2—C9	122.3 (3)	C17—C18—H18B	109.5
C3—C4—C5	120.0 (4)	H18A—C18—H18B	109.5
C3—C4—H4	120.0	C17—C18—H18C	109.5
C5—C4—H4	120.0	H18A—C18—H18C	109.5
C1—C6—C5	116.2 (4)	H18B—C18—H18C	109.5
C1—C6—C7B	132.9 (8)		

### Hydrogen-bond geometry (Å, °)

**Table:**

*D*—H···*A*	*D*—H	H···*A*	*D*···*A*	*D*—H···*A*
N1—H1A···O6	0.89	2.41	3.029 (4)	127
N1—H1B···O4	0.89	2.27	3.043 (5)	146
N1—H1B···O2^i^	0.89	2.48	2.889 (4)	109
N1—H1C···O3^ii^	0.89	2.10	2.935 (4)	157
N1—H1C···O2^i^	0.89	2.58	2.889 (4)	101
N2—H2A···O1	0.89	2.17	2.971 (4)	149
N2—H2B···O5	0.89	2.39	2.875 (4)	114
N2—H2B···O7^iii^	0.89	2.24	2.991 (4)	141
N2—H2C···O7^iv^	0.89	2.03	2.805 (3)	144
C3—H3···O3^v^	0.93	2.60	3.321 (5)	134
C13—H13···O8^vi^	0.93	2.49	3.418 (6)	172

Symmetry codes:  (i) *x*, −*y*+3/2, *z*−1/2; (ii) *x*, *y*, *z*−1; (iii) *x*, −*y*+3/2, *z*+1/2; (iv) *x*, *y*, *z*+1; (v) −*x*, −*y*+1, −*z*+1; (vi) −*x*+1, −*y*+1, −*z*+1.
